# Short‐term effects of NTI‐tss and Michigan splint on nocturnal jaw muscle activity: A pilot study

**DOI:** 10.1002/cre2.371

**Published:** 2020-12-25

**Authors:** Nenad Lukic, Timo Saxer, Mei‐Yin Hou, Aleksandra Zumbrunn Wojczyńska, Luigi M. Gallo, Vera Colombo

**Affiliations:** ^1^ Clinic of Masticatory Disorders, Center of Dental Medicine University of Zurich Zurich Switzerland

**Keywords:** bruxism, Electromyography, oral appliance, TMD

## Abstract

**Objectives:**

Sleep bruxism is mostly assessed by reporting of tooth‐grinding or clenching during sleep and by clinical signs (tooth wear, cracks, or fractures). Parafunctional tooth damage is usually prevented by employing occlusal appliances mainly of the full arch covering type (Michigan splint) and of the partial type covering only central incisors (NTI‐tss). To date, the effects of occlusal appliances on sleep bruxism or jaw muscle activity during sleep are still controversial. The present study is a randomized controlled clinical trial that evaluated the effects of two different splint designs on jaw muscle activity in sleep bruxers otherwise healthy.

**Material and Methods:**

Ten patients from a private dental practice were treated by a single operator. A Michigan splint and an NTI‐tss device were manufactured individually and used at random order. Electromyographic jaw muscle activity was recorded for four consecutive nights in the first, fourth, and seventh week with and without splint. Participants reported on splint comfort and side effects.

**Results:**

Muscle activity decreased only while wearing the NTI‐tss device. Most patients preferred though the Michigan splint due to its greater wearing comfort.

**Conclusions:**

NTI‐tss devices proved more effective for the reduction of jaw muscle activity during sleep. The main advantage of the prefabricated NTI‐tss is its prompt availability in an acute phase of temporomandibular disorders associated with sleep bruxism. In long‐term therapies, patients should be informed of the possible risk of irreversible occlusal changes. Subjective preferences, wearing comfort, and costs should also be considered.

## 1 INTRODUCTION

Bruxism describes masticatory muscle activities that occur during sleep (characterized as rhythmic or non‐rhythmic) and wakefulness (characterized by repetitive or sustained tooth contact and/or by bracing or thrusting of the mandible), respectively (Lobbezoo et al., 2018). The prevalence of sleep bruxism in the general population is estimated to be around 13% (Manfredini, Winocur, et al., 2013). However, the numbers vary widely, depending on the diagnostic tools applied and the study population chosen (Manfredini, Restrepo, Diaz‐Serrano, Winocur, & Lobbezoo, 2013a; Soares et al., 2017).

The etiology of sleep bruxism is still inconclusive, including intrinsic and extrinsic, psychological, or pathophysiological factors often resulting from multiple causes (Kuhn & Türp, 2018; Lobbezoo & Naeije, 2001). Emotional stress and anxiety are often associated with bruxism, as well as an increased level of aggression (Ahlberg et al., 2002; Alves et al., 2013; Molina & Santos, 2002). Morphological factors, such as dental occlusion, were historically discussed as possible causes of bruxism. However, recent studies have shown no such association. Therefore, this explanation can be considered obsolete (Lavigne et al., 2008; Lobbezoo et al., 2012), thus confirming the hypothesis of a centrally regulated mechanism, as suggested by Lobbezoo and Naeije in 2001, also stating that bruxism is “part of an arousal response” and that disturbances in the dopaminergic system may cause sleep bruxism (Lobbezoo & Naeije, 2001). Psychotropic drugs affecting dopamine and serotonin neurotransmission, as well as smoking, consuming caffeine, and heavy alcohol drinking, may also induce bruxism (Lavigne et al., 2008; Lobbezoo et al., 2014).

For the diagnosis of sleep bruxism, Lobbezoo et al. suggested a grading system, subdividing sleep or awake bruxism in “possible,” “probable,” and “definite.” “Possible” sleep/awake bruxism is based merely on the patients' self‐report and/or a questionnaire, whereas “probable” sleep/awake bruxism is additionally based on a clinical examination. A diagnosis of “definite” sleep/awake bruxism must be confirmed by a clinical examination and a polysomnographic recording (Lobbezoo et al., 2013).

The standard approach for the management of sleep bruxism is the use of an occlusal appliance (OA), wholly or partially covering the dental arch. However, no consensus has been reached so far about the mechanisms of action of OAs. It is still unclear whether OAs lead to an increase in muscle activity, a decrease, or no change at all. For this reason, the main purpose of OAs still lies in protecting the teeth, rather than influencing muscle activity during sleep (Jokubauskas et al., 2018).

For the management of sleep bruxism, several splint types are available. One type is the Michigan splint. It covers the whole dental arch (usually the maxillary) with each opposing tooth making one contact to the splint plane. Canine guidance is aimed for lateral and protrusive movements (Geering & Lang, 1978).

Another commercially available and often used type of splint is the “nociceptive trigeminal inhibitory tension suppression system” (NTI‐tss). Approved in 1998 by the American Food and Drug Administration, it has been in use since then. According to its distributor, it is indicated for the management of bruxism, temporomandibular disorders, migraine, and migraine‐associated tension‐type headaches (Karr Dental, 2019a). It consists of a prefabricated shell covering the central maxillary incisors, while evenly contacting the opposing mandibular central incisors. During jaw closure, lateral, and protrusive movements only the lower incisors contact the NTI‐tss device (Karr Dental, 2019a).

This study aimed to compare the effects of a Michigan splint and an NTI‐tss device on the activity of the masseter muscle in patients diagnosed with sleep bruxism by means of electromyography (EMG). The null hypothesis was that the nocturnal EMG activity of the masseter muscle would not be influenced by wearing either a Michigan splint or an NTI‐tss device.

## 2 MATERIAL AND METHODS

This randomized controlled clinical trial was conducted on patients diagnosed with sleep bruxism, referred from a private practice in Switzerland to the study investigators at the Clinic of Masticatory Disorders of the University of Zurich between May and August 2017. The study was performed in accordance with the Declaration of Helsinki on medical protocol and ethics and was approved by the local ethics committee (EKNZ; resolution no. 2017‐00999).

Inclusion criteria were: probable sleep bruxism diagnosis (according to the grading system by Lobbezoo et al., 2013), age between 18 and 50, no current myofascial pain and no previous splint therapy, willingness to participate in the study. Patients with a pathological dental or periodontal condition, planned or ongoing orthodontic treatment, regular use of medical drugs (muscle relaxants, analgesics, anti‐depressants, opioids), and sleeping or neurological disorders were excluded. The diagnosis of probable sleep bruxism was based on the patients' self‐report, confirmation by sleeping partners, and clinical examination. The clinical examination was performed according to the Diagnostic Criteria for Temporomandibular Disorders (DC/TMD) by the International network for orofacial pain and related disorders methodology (INfORM; International Network for Orofacial Pain and Related Disorders Methodology, 2019). A convenience sample of 10 participants (4 males and 6 females, 30 ± 6 years old) was selected for this preliminary study. Each participant gave written informed consent after being adequately informed by the investigators and before starting the study.

**FIGURE 1:**
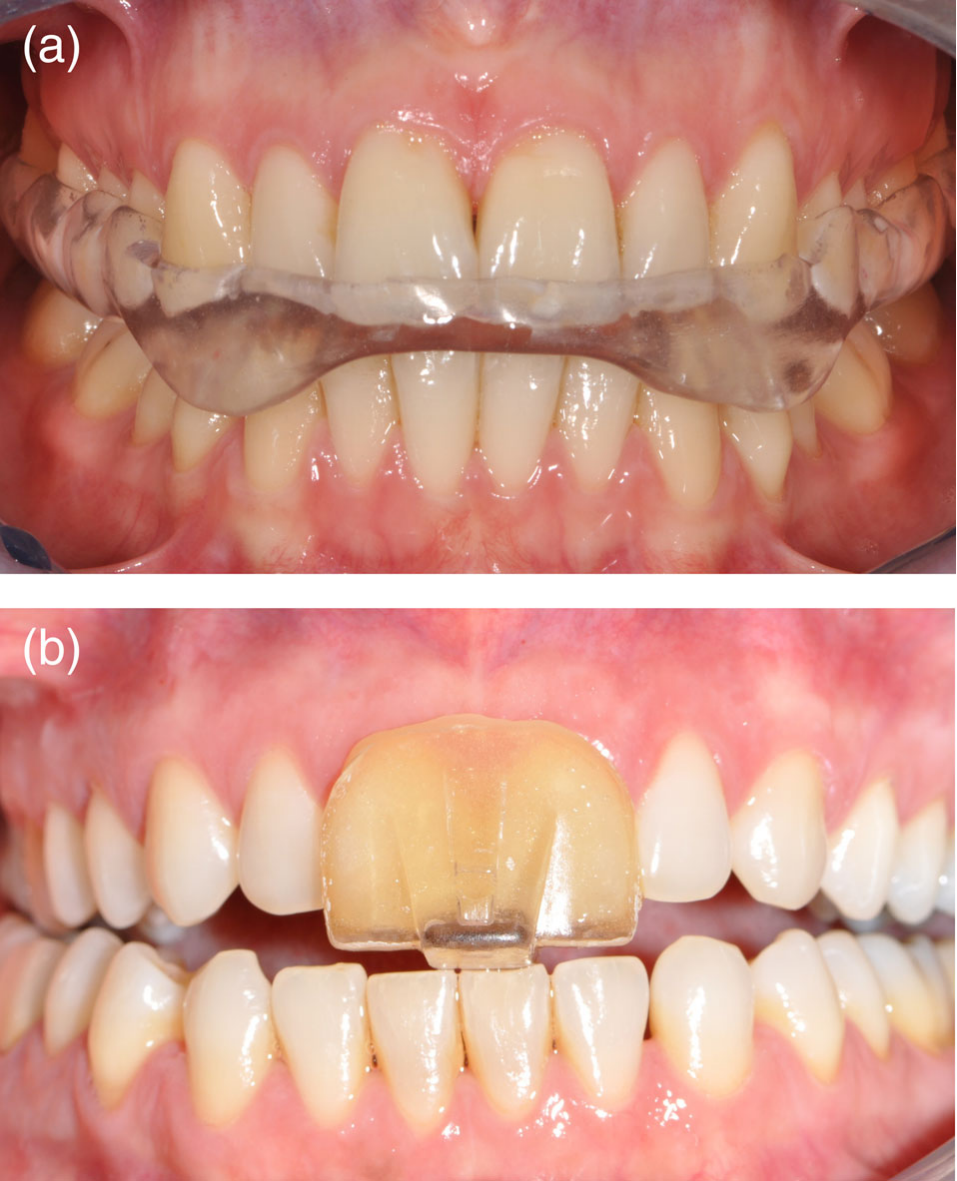
Examples of the splints fabricated for each participant of the clinical trial. (a) Full arch covering splint (Michigan splint) and (b) anterior covering device NTI‐tss

A Michigan splint and an NTI‐tss device were fabricated for each participant. The Michigan splint was manufactured using computer‐aided design/computer‐aided manufacturing (CAD/CAM). First, the upper and lower dental arches of each subject were scanned using an intraoral scanner (TRIOS, 3Shape A/S, Copenhagen, Denmark), then the splint was digitally designed for the maxilla, according to the standard features (evenly distributed occlusal contacts from the lower jaw teeth, canine guidance during protrusion and laterotrusion) through CAD software for dental applications (Ortho Analyzer™, Kulzer, Hanau, Germany; Appliance Designer™, 3Shape A/S, Copenhagen, Denmark). The splint was then produced from a blank of Poly(methyl Methacrylate) (PMMA) (DD Bio Splint P HI Dental Direkt, Spenge, Germany), a hard acrylic resin, using a milling machine (inLab MC X5, Dentsply Sirona, York) (Figure 1(a)). The NTI‐tss device was made covering the maxillary incisors and contacting only the tips of the lower middle incisors, according to the manufacturers' guidelines (Karr Dental, 2019b; Wollerau, Switzerland). It was positioned so that its occluding plane was parallel to the opposing arch plane and perpendicular to the lower incisors. Once the correct position had been found, the shell was filled chair‐side with an auto polymerizing acrylate for adjustment to the patient's teeth (Figure 1(b)). After the material had settled, the outer surface was adjusted and polished.

To track the bruxism activity of the patients, EMG signals of the right masseter muscle were acquired during sleep. Custom‐made portable EMG recorders (BioSignal Recorder, University of Zurich, Switzerland) were used (Gallo et al., 1999) for the acquisition of reliable data from each patient in their habitual sleep environment, These devices carried a LED display, two acquisition channels, a keyboard, a cable for electrodes connection, and a memory card for data storage. The sampling frequency was 2 kHz. To optimize the signal‐to‐noise ratio, the differential EMG signal was amplified 4'000×, band‐pass filtered between 50 and 1000 Hz, and 10 bit‐digitized (Čadová & Gallo, 2014). Signals were saved in Waveform Audio File Format (.WAV).

The patients were instructed to place two self‐adhesive pre‐gelled disposable Ag‐AgCl rectangular surface electrodes (Ambu® Neuroline 720, Ambu A/S, Ballerup, Denmark), measuring 30 × 22 mm, on the right masseter muscle, and one reference electrode on the skin over the right mastoid process before going to bed. The skin over the masseter muscle and the mastoid process was prepared before applying the electrodes, according to the recommendations of the SENIAM project (Surface Electromyography for the Non‐invasive Assessment of Muscle, Enschede, the Netherlands). The skin was rubbed with abrasive paste and water (Lubex peeling®, Permamed AG, Therwil, Switzerland) and cleaned with an electrode solution for skin preparation (Signaspray®, Parkerlabs, Fairfield) to ensure a sufficient quality of the EMG signal. Male patients were advised to shave daily during the recording periods to improve the adhesion of the electrodes. Each patient was briefly instructed on how to use the EMG recorder to avoid technical problems during the recording.

**FIGURE 2:**
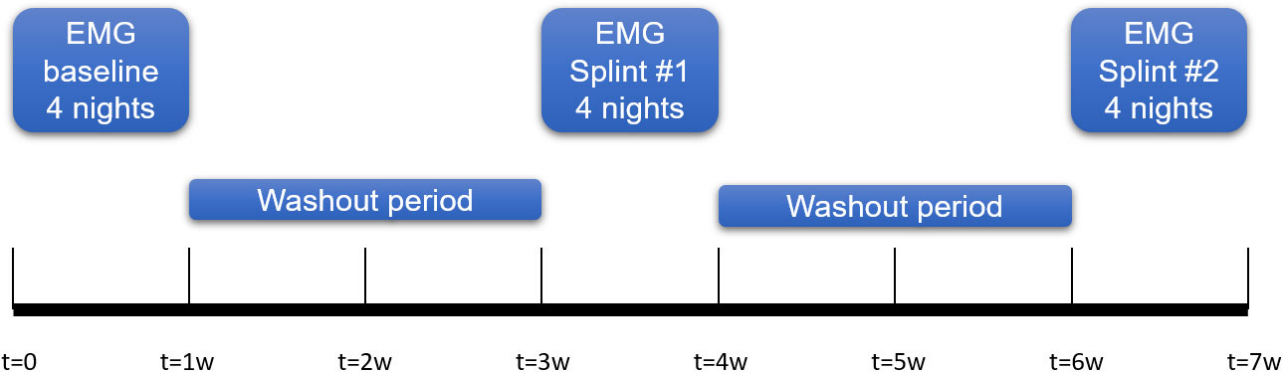
Timeline of the study protocol. The first electromyography recording session was a baseline recording without an occlusal splint (*t* = 0 to *t* = 1 week). For the second recording session (*t* = 3 to *t* = 4 weeks) the patients wore one of the two splints and for the third recording session they wore the other splint (*t* = 6 to *t* = 7 weeks). A washout period of 2 weeks was observed between the recording sessions (*t* = 1 to *t* = 3 weeks and *t* = 4 to *t* = 6 weeks). The order in which the patients wore the splints was randomized

The study protocol for each patient lasted 7 weeks in total (Figure 2). EMG recordings were conducted in three sessions of four consecutive nights. In each session, a different condition was tested. The first four nights of EMG recording were performed as baseline without occlusal splint, followed by a washout period of 2 weeks. In the next two sessions each patient wore, in a randomized order, either the Michigan splint or the NTI‐tss device during four consecutive nights, followed by a wash‐out period of 2 weeks, and then the other splint. The splint order was randomized with Microsoft Excel (MS Excel 2016). At the beginning of each recording, the patients were instructed to clench three times at their maximum effort for 3 s at a 5‐s interval for the acquisition of the signal of the maximum voluntary contraction (MVC) of their masseter muscles. Each patient was requested to write a sleep diary, listing the approximate time they fell asleep, and the time they woke up including all sleep disturbances they experienced during the night (e.g., awake time, drinking, eating, etc.). This information was needed to remove the corresponding time intervals from the EMG recording in the signal analysis and thus avoid confounding factors. In return for participating in the trial, each patient received the Michigan splint and the NTI‐tss device free of charge. At study completion, the patients returned to the dental practice for the regular follow‐ups to control the splint fit, to manage their sleep bruxism in general, and to prevent possible side effects due to the NTI‐tss device. Each participant was asked two open questions: (1) Which splint did you prefer to wear? and (2) Did you experience any problem in adjustment and comfort while wearing the splint? The answers were noted by the operator.

Before EMG analysis, all acquired signals were visually inspected to detect noise artefacts using an open‐source audio‐editing software (Audacity®, n.d.; Version 1.3). If necessary, the signal was edited for the elimination of the periods listed in the sleep diary to correctly detect muscle activities only during sleep. The first 30 min and the last 10 min of each recording were excluded from the analysis, estimated as the time needed to fall asleep and waking up.

Recordings were analyzed by a custom‐made electromyography analysis tool (EMGAT) developed at the Laboratory of Physiology and Biomechanics of the University of Zurich (Čadová & Gallo, 2014). Signals higher than a cutoff threshold of 10% MVC with a minimum duration of 0.25 s and separation time of 5 s were detected and defined as single muscle activities. The thresholds were set according to the literature (Gallo et al., 1999; Lavigne et al., 1996; Omoto et al., 2015). Afterward, the number of activities per hour (NA/h) and the average duration of the activities (AvgDurAct) (s) were determined, and the average amplitude of the activities (AvgAmplAct) was calculated as a percentage of the MVC (% MVC). Descriptive statistics was computed for all the variables within the observed conditions (baseline, Michigan, and NTI‐tss). Statistical tests were performed for the parameters obtained from the EMG recordings always comparing the baseline to the NTI‐tss device and the Michigan splint. A linear mixed effect model was applied to the data. A random intercept was used to eliminate inter‐individual differences of the patients and a fixed effect for each therapy was used to determine the influence of the therapy on each patient after elimination of inter‐individual differences. The influence of the Michigan splint and the NTI‐tss device on the variables NA/h, AvgDurAct, and AvgAmplAct was then calculated. All statistical analyses were performed using the free statistical software environment R Version 3.5.1 (R. Core Team, 2013) including the packages ggplot2 (Wickham, 2016) and lmerTest (Kuznetsova et al., 2017). The level of significance was set at α = 0.05. Power analysis with power set at β = 0.8 was performed to calculate the optimal sample size (IBM SPSS Software V.23 for Windows and GLIMMPSE) (Guo et al., 2013).

## 3 RESULTS

All 10 patients could complete the required measurements. No adverse events occurred during the study period. Seven patients preferred to wear the Michigan splint since they felt it more comfortable. Remarks on the NTI‐tss device: one patient reported an increased salivary flow, one patient experienced pain in the lower incisors after each night, one patient preferred the NTI‐tss device because of its smaller size compared to the Michigan splint. None of the patients had any problems tolerating the splints during the night. Of all recordings, four nights could not be analyzed due to technical reasons (e.g., electrode disconnection) and they were treated as missing data. In total, 116 EMG‐recordings were obtained.

**FIGURE 3:**
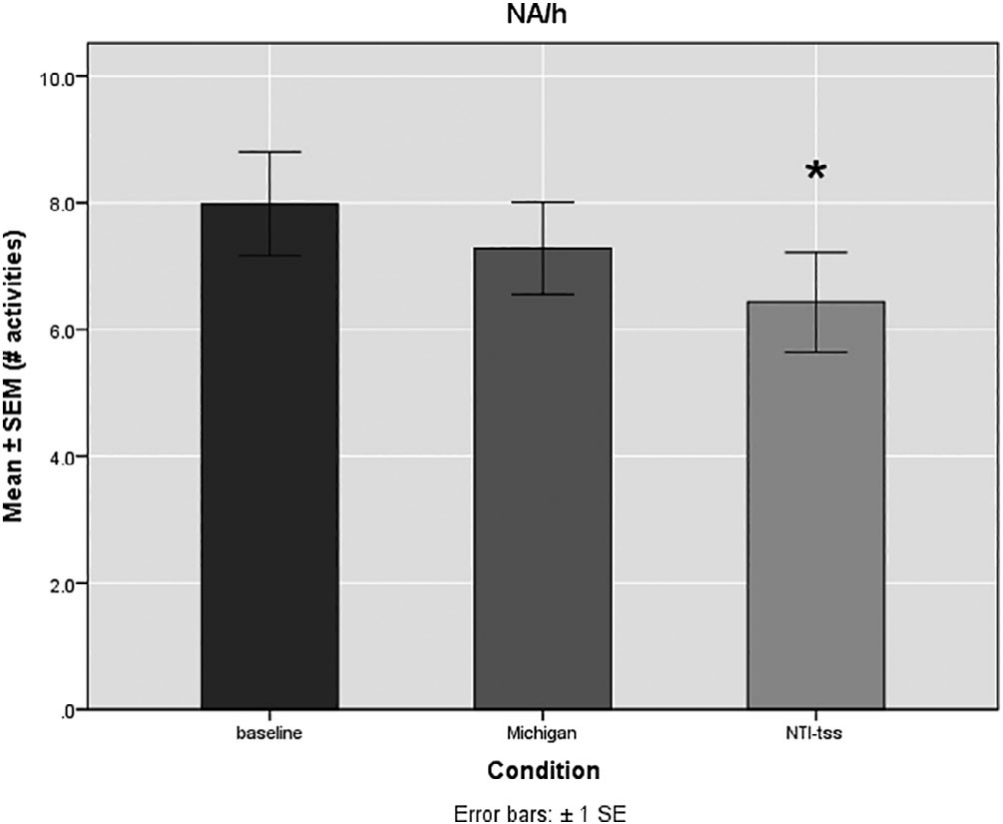
Mean number of activities per hour (NA/h) ± SE of the mean (SEM) for the observed conditions (Baseline, Michigan, NTI‐tss). The baseline value was 8.1 ± 1.4 NA/h. The NTI‐tss devices led to a significant reduction in the NA/h (6.4 ± 0.6) whereas the Michigan splints showed no significant effect (7.3 ± 0.6). *p = 0.004; *n* = 10

**FIGURE 4:**
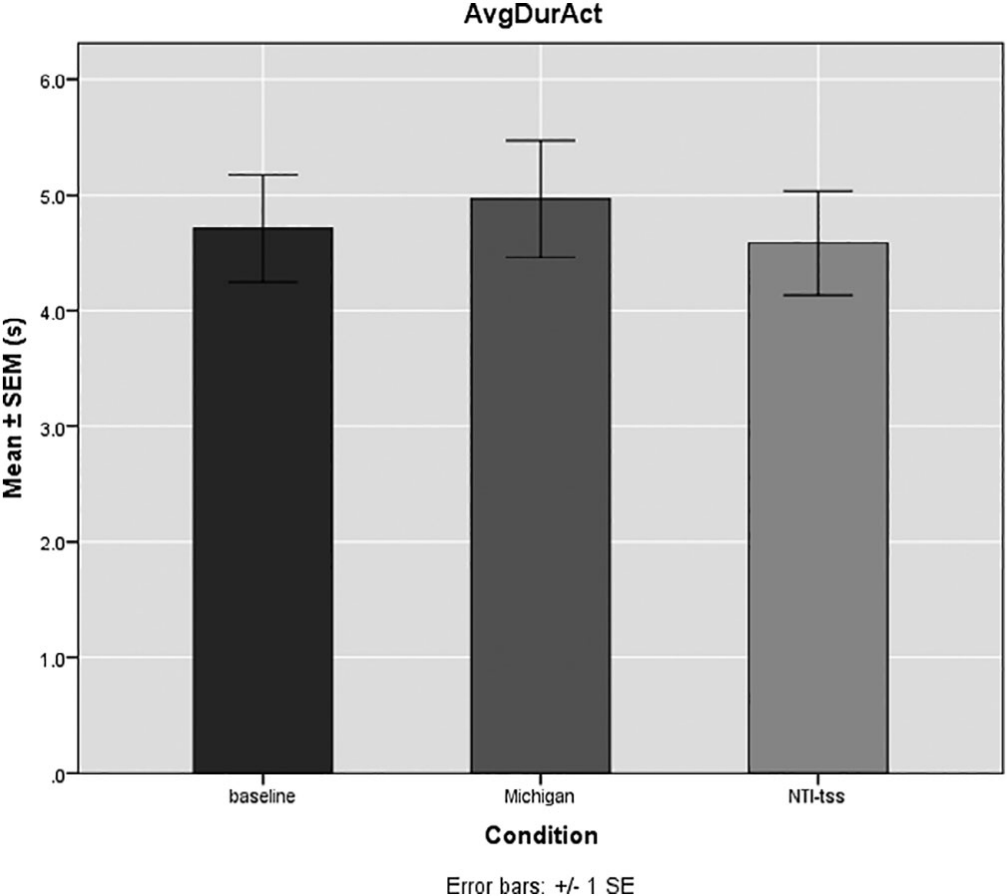
Mean average duration of the activities (AvgDurAct) ± SE of the mean (SEM; s) for the observed conditions (baseline, Michigan, and NTI‐tss). The baseline value of the average duration of the activities was 4.7 ± 0.6 s. No significant differences in the average duration of the activities were observed for both splints compared to baseline. The Michigan splints showed a tendency to increase the average duration of the activities compared to baseline (5 ± 0.6 s; *n* = 10)

**FIGURE 5:**
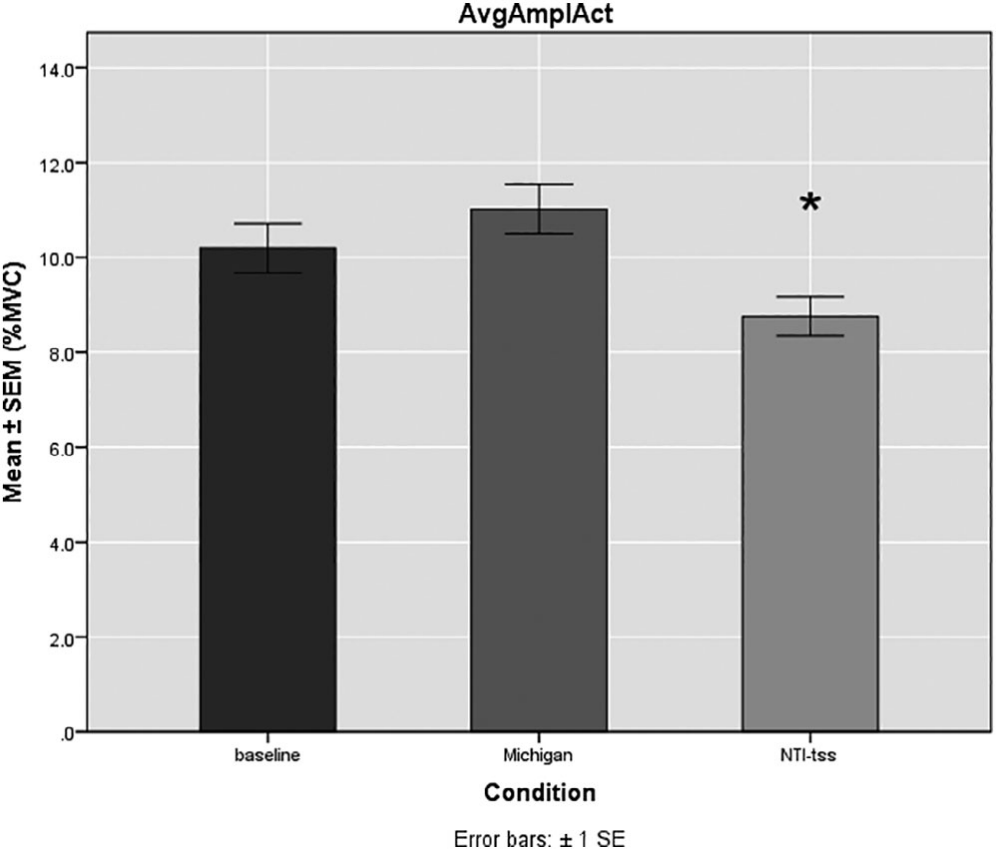
Mean average amplitude of the activities (AvgAmpAct) ± SE of the mean (SEM; %MVC) for the observed conditions (baseline, Michigan, NTI‐tss). The baseline value was 10.3 ± 0.7. The NTI‐tss splints led to a significant reduction in the average amplitude of the activities (8.7 ± 0.6) whereas the Michigan splints showed a tendency to increase the average amplitude of the activities (11 ± 0.6; *p = 0.01; *n* = 10)

The baseline value of the number of activities per hour was 8.1 ± 1.4 (mean ± SE of the mean). The average duration of the activities at baseline was 4.7 ± 0.6 s. The average signal at baseline was 10.3 ± 0.7 %MVC. Statistical analysis showed that the Michigan splint did not significantly reduce the number of activities per hour when compared to baseline (7.3 ± 0.6; *p* = 0.165), whereas the NTI‐tss device led to a significant reduction of the number of activities per hour (6.4 ± 0.6; *p* = 0.004) (Figure 3). Neither device influenced significantly the average duration (s) of the activities. Interestingly, the Michigan splint rather lengthened the average duration of the activities (5 ± 0.6 s; *p* = 0.66) (Figure 4). The average signal measured in % of the MVC was significantly reduced only by the NTI‐tss device (8.7 ± 0.6 %MVC; *p* = 0.01). The Michigan splints slightly increased the signal of the activities compared to baseline, but with no statistical significance (11 ± 0.6 %MVC; *p* = 0.211) (Figure 5).

## 4 DISCUSSION

The present study compared the effects of two different splint designs on the EMG activity of the masseter muscle during sleep in a group of bruxers without other signs of TMD. The Michigan splint was selected as gold standard because it is commonly used in the management of sleep bruxism. The NTI‐tss device was chosen because it is often used in private practice and cost‐ and fabrication‐effective but it is still controversial, due to its potential side effects (Stapelmann & Türp, 2008). A hard material was used for splint fabrication since it has been proven to reduce jaw muscle activity more effectively than soft material (Okeson, 1987).

The main findings observed are the intra‐individual difference in masseter activation during sleep between baseline and wearing an NTI‐tss device. Indeed, the latter led to a significant drop in the number of activities per hour (NA/h) and a significant reduction of the average signal of the activities (AvgAmplAct). However, the average duration of the activities (AvgDurAcr) did not show to be significantly influenced by wearing the NTI‐tss. This effect could be observed throughout the wearing time of four nights. Furthermore, this result seems to be consistent inter‐individually in our sample.

The use of a Michigan splint, on the contrary, did not lead to a significant reduction of the three observed parameters compared to baseline, as well as to the use of an NTI‐tss device. In this condition, only a trend toward a reduction of the number of activities per hour and the average signal of the activities could be observed. Conversely, a slight increase (although not significant) of the activity duration and the average amplitude of the activities was noticed. Similar results on both Michigan and NTI‐splints were found in a clinical trial by Baad‐Hansen et al. (2007), who reported a significant decrease in the number of masseter activity per hour while wearing the NTI‐tss device, but not with another occlusal splint type. However, in their work, a decrease in the duration of EMG activity was found as well. Two other trials investigating the effects of occlusal devices also found no reduction of masticatory muscle activity when wearing an occlusal splint (Muhtaroğulları et al., 2013; van der Zaag et al., 2005) while other authors reported an activity reduction when wearing an OA (Hiyama et al., 2003; Solberg et al., 1975).

The significant reduction of the activity amplitude when wearing the NTI‐tss device is also in accordance with the literature (Muhtaroğulları et al., 2013). This result could be attributed to an increase in the activation of periodontal mechanoreceptors. In fact, the hypothesized mechanism of action of the NTI‐tss device is based on the different neuronal feedback between incisor and posterior teeth. For all teeth, the periodontal mechanoreceptors are providing information about tooth load to the central nervous system. These mechanoreceptors are sensitive to static and rapidly changing forces occurring also during parafunctional movements. The receptors surrounding incisor teeth show a much higher sensitivity at low force levels compared to the receptors of posterior teeth (Johnsen & Trulsson, 2005). When biting on the NTI‐tss device with the incisors, the periodontal mechanoreceptors in the area of the loaded teeth accordingly already react to lower forces. It is likely that the input from the receptors is modulating jaw muscle activity (Trulsson & Johansson, 1996), that is, that overexertion of periodontal mechanoreceptors leads to a reflex inactivation of masticatory muscles and a reduction of muscle tension. One result supporting this theory indirectly is that, in the present investigation, Michigan splints did not lead to such a reduction. This differential effect of a splint covering the whole dental arch and an NTI‐tss device on the activity amplitude was also observed in other studies (Muhtaroğulları et al., 2013).

The lack of statistical significance in the decreased duration of the activities in our study may be attributed to the small sample size and the short wearing period (3 × 4 nights). Indeed, a power analysis showed that 31 subjects would be required for sufficient power (β = 0.80) to detect significant differences in the variable considered. However, the finding that the duration of the activities was not influenced by the NTI‐tss device is consistent with another study conducted in 2013 (Muhtaroğulları et al., 2013). Still, the topic remains controversial.

Previous authors reporting on NTI‐tss found the device not superior to a standard occlusal splint in the management of TMDs. However, their focus lay on signs and symptoms of TMD and therapy outcome and did not analyze EMG values of jaw muscle activity during the night (Lobbezoo et al., 2013; Magnusson et al., 2004), whereas others found no association between reduction of EMG activity and reduction of TMD signs and symptoms. Indeed, for the choice of a device for the management of sleep bruxism, not only the effectiveness in reducing jaw muscle activity should be considered. It is also crucial to include in the decision‐making process costs, wearing comfort, and possible side effects.

Despite all efforts to provide a qualitatively high research investigation, limitations were encountered during data collection, processing and statistical analysis. One limitation of this study lays in EMG data processing. Indeed, it is known that mimic muscles may influence recorded EMG activities, making it difficult to distinguish between facial muscles and masseter muscle. To eliminate this interference, the activity cut‐off threshold was set at 10%. This threshold is widely used in the literature (Baad‐Hansen et al., 2007; van der Zaag et al., 2005). However, this elimination might lead to an underestimation of the real masseter activities. Indeed, to eliminate selectively spurious EMG activities (background noise, mimic muscle activity, etc.), a polysomnographic test should have been conducted, since polysomnography remains the gold standard to verify sleep bruxism (Lobbezoo et al., 2013). Due to high expenditure and costs, the disadvantage of sleeping in a laboratory set‐up, and the limited wearing period, polysomnographic recordings would not have been justified for this preliminary study. Another methodological issue that might have influenced the results is the chronological order in which baseline recordings were set. We chose to conduct baseline EMG‐recordings before the other recordings since none of the patients had experienced an occlusal splint before, following protocols found in the literature (Baad‐Hansen et al., 2007). For statistical reasons, the EMG measurement without splint should have been randomized, or further 4 days of EMG measurement without splint should have been scheduled after the completion of the study. Finally, the small sample size of this work could give rise to statistical errors. In order to verify the effect of the NTI‐tss device on the number of activities per hour and the activity amplitude, the number of patients should be increased. Likewise, the sample size should be increased to verify the parameters for which no significant difference was observed for either splint.

Especially for the NTI‐tss device, there are reporting of unwanted side effects, for example, occlusal changes, aspiration or ingestion of the device, increased mobility of anterior teeth, or hypersensitivity of lower front teeth (Jokstad et al., 2005; Magnusson et al., 2004). The side effects of the NTI‐tss device affecting occlusal changes can be minimized when the splint is only worn during sleep and only for a short time (Stapelmann & Türp, 2008). For this reason, besides the data on EMG activity, the comfort and side effects were also documented during the trial. One of the patients reported increased salivation while wearing the NTI‐tss device. This change in saliva flow is well known in all kinds of OAs but assessed as “usually short‐lasting” (Stapelmann & Türp, 2008). Another patient experienced pain in the lower front teeth after wearing the NTI‐tss device, thus confirming reports of this unwanted side effect. None of the patients reported the loosening or falling out of the NTI‐tss device during sleep, which has otherwise been reported by other authors (Jokstad et al., 2005). Seven of ten patients reported a lower comfort when wearing the NTI‐tss device, whereas no occlusal changes occurred in any patient since trial completion: as suggested in the literature, we recommend to wear the splint only during sleep (Stapelmann & Türp, 2008).

Finally, although statistical analysis demonstrated a significant decrease in the activation frequency and in the signal amplitude of the masseter muscle after insertion of an NTI‐tss device, the question remains whether this change might have a clinical relevance in localized TMD and contribute to an improvement of patients' condition. Furthermore, this result is by no means considered as the sole parameter for selecting oral appliances for bruxism therapy. Together with the objective decrease of muscle activity, a profound knowledge about the risks of the available devices and an assessment of benefits for the patient are fundamental in the choice of the type of appliance.

## 5 CONCLUSION

The data of this pilot study indicate the possibility that NTI‐tss device may be equally or more effective than the Michigan splint in reducing jaw muscle activity during a period of four nights. The NTI‐tss device is prefabricated, easily available, and quickly adapted in a dental practice. Besides an objective reduction of muscle activation, however, for frequent occurrence or as a long‐term therapy, one should as well consider the subjective wearing comfort—which seems to be impaired while wearing the NTI‐tss device—and of course the risk of irreversible occlusal changes. Whether the price for an acute phase therapy is justified should also be included in the decision, since bruxism accompanies patients over a longer period of time. Further studies with larger samples and longer therapy duration are needed to obtain more conclusive data for clinical application.

## Data Availability

Data available on request due to privacy/ethical restrictions
